# Thirty Years Later: Evolution of Treatment for Acute Left Main Coronary Artery Occlusion

**DOI:** 10.1155/2016/7360682

**Published:** 2016-12-19

**Authors:** Moshe Y. Flugelman, Nissan Ben-Dov, Basheer Karkabi, Ronen Jaffe

**Affiliations:** Department of Cardiovascular Medicine, Lady Davis Carmel Medical Center and the Ruth and Bruce Rappaport Faculty of Medicine, Technion, Israel Institute of Technology, Haifa, Israel

## Abstract

Acute occlusion of left main coronary artery is a catastrophic event. We describe two patients with acute occlusion of the left main coronary artery treated thirty years apart. The first patient was treated in 1982 and survived the event without revascularization but developed severe heart failure. His survival was so unusual that it merited a case report at that time. The second patient was treated at the end of 2015. Early revascularization resulted in myocardial reperfusion and near normal left ventricular function. These patients exemplify the progress in therapeutic cardiology over the last 30 years.

## 1. Introduction

Thirty-three years ago we treated a 55-year-old man that presented with half an hour of chest pain and developed ventricular fibrillation. The patient was resuscitated but developed cardiogenic shock. Later he was found to have left main coronary artery (LMCA) occlusion. He survived the event after long hospitalization and developed severe heart failure due to poor left ventricular function [1]. Recently we treated a 62-year-old woman that presented with severe chest pain and cardiogenic shock due to acute left main coronary occlusion. The patient was catheterized shortly after arrival, stent was placed in her LMCA, and after 14 days of hospitalization she was discharged with nearly normal left ventricular function. Thirty years ago survival after acute LMCA occlusion was rare and merited publication of a case report [1]; it is still uncommon; nonetheless the following two cases illustrate the progress made in cardiology over the last 30 years [2, 3].

## 2. Case Reports

### 2.1. Patient Number 1, 1982

**Figure 1:**
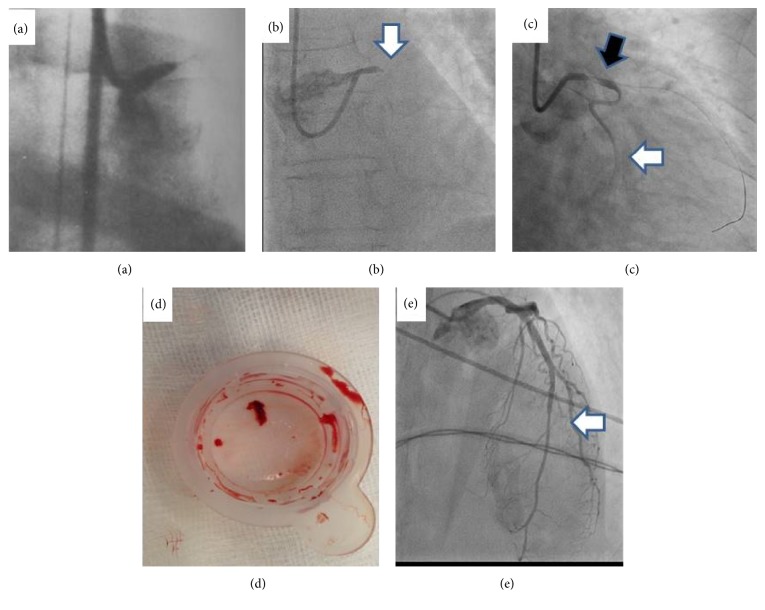
(a) Coronary angiography of patient number 1, catheterized 15 days after arrival showing complete occlusion of the left main coronary artery. Note femoral approach (published with permission from Elsevier), (b) patient number 2 coronary angiography showing complete occlusion of the left main coronary artery (white arrow), (c) after passing a guidewire down the left main and left anterior descending arteries the circumflex coronary artery was demonstrated (white arrow); left main coronary artery filling defect is demonstrated (black arrow), (d) thrombus aspirated from the left main prior to angioplasty, and (e) patient left main and left anterior descending coronary arteries demonstrated during the second catheterization, three days after arrival.

A 55-year-old man, heavy smoker with no history of ischemic heart disease, developed ventricular fibrillation 30 minutes after the onset of severe retrosternal chest pain. Sinus rhythm was restored after 6 direct-current cardioversions combined with lidocaine infusion. After resuscitation, pulmonary edema developed; the patient had a systolic blood pressure of 60 mm Hg, a pulse rate of 120 beats/min, and frequent premature ventricular contractions. Electrocardiogram showed sinus tachycardia with frequent ventricular premature contractions and an acute extensive anterior myocardial infarction. The cardiac index, measured with a thermodilution Swan-Ganz catheter, was 1 liter/min/m^2^. An intra-aortic counterpulsation balloon (IACPB) was inserted through the right femoral artery and blood pressure increased to 110/70 mm Hg and the cardiac index to 1.9 liters/min/m^2^. Mechanical assistance was combined with intravenous treatment of glucose-potassium-insulin solutions, oral isosorbide-dinitrate, digoxin, and diuretic agents. Cardiac catheterization and angiography, performed 15 days after admission, revealed a left ventricular end-diastolic pressure of 35 mm Hg, a large aneurysm of the anterior and apical regions, and a normal contraction of the inferior wall. Coronary arteriography demonstrated complete obstruction of the LMCA (Figure 1(a)) while the right coronary artery was normal. There was no collateral filling of the left coronary system. The left ventricular ejection fraction was 16% with mechanical assistance and 5% without assistance. After 26 days, the patient developed a shaking chill and the IACPB was removed. The patient was discharged after 40 days. By this time he had signs of moderate congestive heart failure but no angina pectoris and was in New York Heart Association functional class III. On discharge, the cardiac index was 1.8 liters/min/m^2^. Radionuclide angiography showed a left ventricular ejection fraction of 10%. A year later he was in functional class III. Three years after the acute event he died of heart failure complications (this case report is a modified version of reference 1 and is published with permission from Elsevier).

### 2.2. Patient Number 2, 2015

A 62-year-old patient with history of smoking and angina on effort for two weeks prior to hospitalization developed severe chest pain and presented at the emergency room in pulmonary edema and blood pressure of 65 mm Hg. Her ECG showed acute myocardial infarction in the anterior wall. The patient was transferred immediately to the catheterization laboratory and normal right coronary artery was demonstrated via the right radial artery. Total occlusion of the left main was demonstrated using a 6F guiding catheter (Figure 1(b)) and a hydrophilic guidewire was passed through the occluded left main coronary artery to the left anterior descending artery (LAD). Immediately after passing the guidewire the circumflex artery was demonstrated (Figure 1(c)). An aspiration device was passed over the wire down the LAD. A red thrombus was aspirated and flow was restored in the LAD (Figure 1(d)). An LMCA stenosis and an LAD stenosis were demonstrated and a drug eluting stent (DES) was placed from the proximal LMCA to the proximal LAD. A second DES was placed in the LAD as LAD distal to the first stent was significantly narrowed. As blood pressure was still low, IACPB was placed via the right femoral artery and the patient was maintained on heparin and dopamine. On days two and three after catheterization the patient had two episodes of pressing chest pain that lasted for 30 minutes and reminded the patient of the symptoms at arrival. Immediately after the second episode that patient had had a second catheterization via the right radial artery which demonstrated TIMI grade III flow in the left coronary system (Figure 1(e)). Echocardiography demonstrated ejection fraction of 50% with mild apical dyskinesia. The patient recovered slowly over the next 10 days and was discharged 12 days after admission.

## 3. Discussion

Over the last three decades treatment of STEMI changed dramatically. The introduction of intravenous thrombolytic therapy and then percutaneous interventions (PCI) reduced in-hospital mortality of young STEMI patients from 30% to <5% [4]. Thirty years ago a patient with STEMI that survived to leave the hospital had had high chances of suffering from significantly reduced left ventricular function and symptoms of heart failure. Nowadays, patients with STEMI that arrive early and are treated with PCI have a better left ventricular function and better prognosis.

Acute occlusion of the left main coronary artery is usually a fatal event, yet 4% of patients that arrive with STEMI and undergo PCI are reported to have left main narrowing and occlusion [5–8]. Reports of survival after acute total occlusion of left main coronary artery were rare prior to the thrombolytic era and therefore the report of the patient we treated in 1982 was published. The patients that we treated in 1982 survived the acute event and left the hospital with ejection function of 10% and NYHA grade III symptoms while the patient treated in 2015 left the hospital with ejection function of 50% and no symptoms. In both patients we used IACPB which is not recommended in current guidelines for treating STEMI patients, yet use of IACPB was a standard practice in STEMI patients with low blood pressure prior to introduction of reperfusion therapies. Although two controlled studies showed no survival benefits of IACPB in STEMI patients, the use of IACPB in the above reported patients contributed to survival of these patients that were presented with low blood pressure and cardiogenic shock [9]. Radial approach is currently the most used approach for coronary catheterization and is associated with fewer complications [10]. As demonstrated in Figures 1(a) and 1(b) the first patient was catheterized from the femoral artery while the second, recent patient was catheterized from the radial artery. Using radial approach required an additional vascular access for the IACPB but, importantly, allows an uninterrupted hemodynamic support from the IACPB during the coronary intervention.

Recommendation for thrombus aspiration in acute STEMI is another component of STEMI treatment that was modified recently. After initial enthusiasm [11] thrombus aspiration use has declined as its effectivity in improving outcomes in STEMI was not proved in large-scale studies [12, 13]. We chose to use aspiration device as large bulk of thrombus was observed after passing the guidewire. Aspiration of a large thrombus can improve visualization but may be associated with thrombus dislodgement to the aorta.

## 4. Conclusion

In summary, conceptual and technical progress has changed the prognosis of STEMI patients in general and in patients with acute LMCA occlusion more specifically as exemplified in the descriptions of the two patients that were treated for the same conditions 30 years apart.
